# Pyruvate kinase M2 phosphorylates H2AX and promotes genomic instability in human tumor cells

**DOI:** 10.18632/oncotarget.22621

**Published:** 2017-11-17

**Authors:** Li Xia, Kang Qin, Xin-Ran Wang, Xiao-Ling Wang, Ai-Wu Zhou, Guo-Qiang Chen, Ying Lu

**Affiliations:** ^1^ Department of Pathophysiology, Key Laboratory of Cell Differentiation and Apoptosis of Ministry of Education, Shanghai Jiao Tong University School of Medicine (SJTU-SM), Shanghai 200025, China

**Keywords:** DNA damage, DNA damage response, pyruvate kinase, γ-H2AX, genomic instability

## Abstract

Pyruvate kinase (PK) catalyzes the conversion of phosphoenolpyruvate and ADP to pyruvate and ATP, a rate-limiting reaction in glycolysis. M2 isoform of PK (PKM2) is the predominant form of PK expressed in tumors. In addition to its well established cytosolic functions as a glycolytic enzyme, PKM2 displays nuclear localization and important nonmetabolic functions in tumorigenesis. Herein, we report that nuclear PKM2 interacts with histone H2AX under DNA damage conditions. Depletion of PKM2 decreased the level of serine 139-phosphorylated H2AX (γ-H2AX) in response to DNA damage. The *in vitro* kinase assay reveals that PKM2 directly phosphorylates H2AX at serine 139, which is abolished by the deletion of FBP-binding pocket of PKM2 (PKM2-Del^515-520^). Replacement of wild type PKM2 with the kinase dead mutant PKM2-Del^515-520^ leads to decreased cell proliferation and chromosomal aberrations under DNA damage conditions. Together, we propose that PKM2 promotes genomic instability in tumor cells which involves direct phosphorylation of H2AX. These findings reveal PKM2 as a novel modulator for genomic instability in tumor cells.

## INTRODUCTION

Cancer cells have increased glucose uptake and lactate production with concomitant decreased oxygen consumption, a phenomenon known as aerobic glycolysis or the Warburg effect. Previous works suggested that expression of pyruvate kinase M2 (PKM2) promotes aerobic glycolysis and plays a critical role in tumorigenesis [1–6]. Pyruvate kinase is the final and rate-limiting enzyme in glycolysis that catalyzes the conversion of phosphoenolpyruvate (PEP) and ADP to pyruvate and ATP. It has been known that PKM gene can express two isoforms by alternative RNA splicing, that is, pyruvate kinase M1 (PKM1) and PKM2 [7]. The highly active PKM1, which forms stable tetramers, is expressed in normal adult tissues such as the heart, brain and skeletal muscle. In contrast, PKM2 can be allosterically regulated by metabolic intermediates such as fructose-1, 6-bisphosphate (FBP) and is convertible between a dimer with low pyruvate kinase activity and a FBP-bound tetramer with high pyruvate kinase activity [8–10]. PKM2 is expressed during embryogenesis and is the predominant form in tumors of different types [11–14]. A large body of evidence supports the notion that PKM2 expression correlates with tumorigenesis and poor prognosis for patients with many kinds of cancers, as reviewed [14]. Mechanistically, it was shown that tumor cells develop multiple post-translational strategies to decrease enzymatic activity of PKM2 including phosphotyrosine-binding, phosphorylation, acetylation and oxidation, leading to the accumulation of glycolytic intermediates for biosynthetic reactions to support proliferation [15–18]. These inhibitory effects on PKM2 activity occur in response to various stimuli that tumor cells may encounter during tumor initiation or maintenance, such as excessive growth factors, high glucose or high reactive oxygen species concentrations [15–18].

More recently, it was noted that PKM2 translocates to the nucleus under certain circumstances such as interleukin-3, epithelial growth factor (EGF), ultraviolet (UV) light and H_2_O_2_ treatment [19–22]. Nuclear PKM2 displays intriguing non-glycolytic functions including phosphorylating some nuclear proteins such as histone H3 [2, 23], signal transducer and activator of transcription 3 (stat3) [24], Bub3 [25], and myosin light chain 2 (MLC2) [26], for which PKM2 uses the high-energy phosphate from PEP but not ATP as a phosphate donor, proposing that PKM2 in cancer cells presents protein kinase activity. More recently, we also reported that the nuclear PKM2 interacts directly with P53 protein and inhibits P53-dependent transactivation of the P21 gene, leading to a nonstop G_1_ phase in cancer cells exposed to DNA-damaging agent [27]. Thus nuclear PKM2 is linked to non-metabolic processes of cancer cells such as proliferation, cell cycle, apoptosis, epithelial-mesenchymal transition and angiogenesis [19, 21, 24, 25, 28-34]. In this report, we show that DNA damage stimulus induces a direct interaction between PKM2 and H2AX. PKM2 phosphorylates H2AX at serine 139, generating γ-H2AX and promotes genomic instability following DNA damage.

## RESULTS

### Nuclear PKM2 interacts with H2AX upon DNA damage

**Figure 1:**
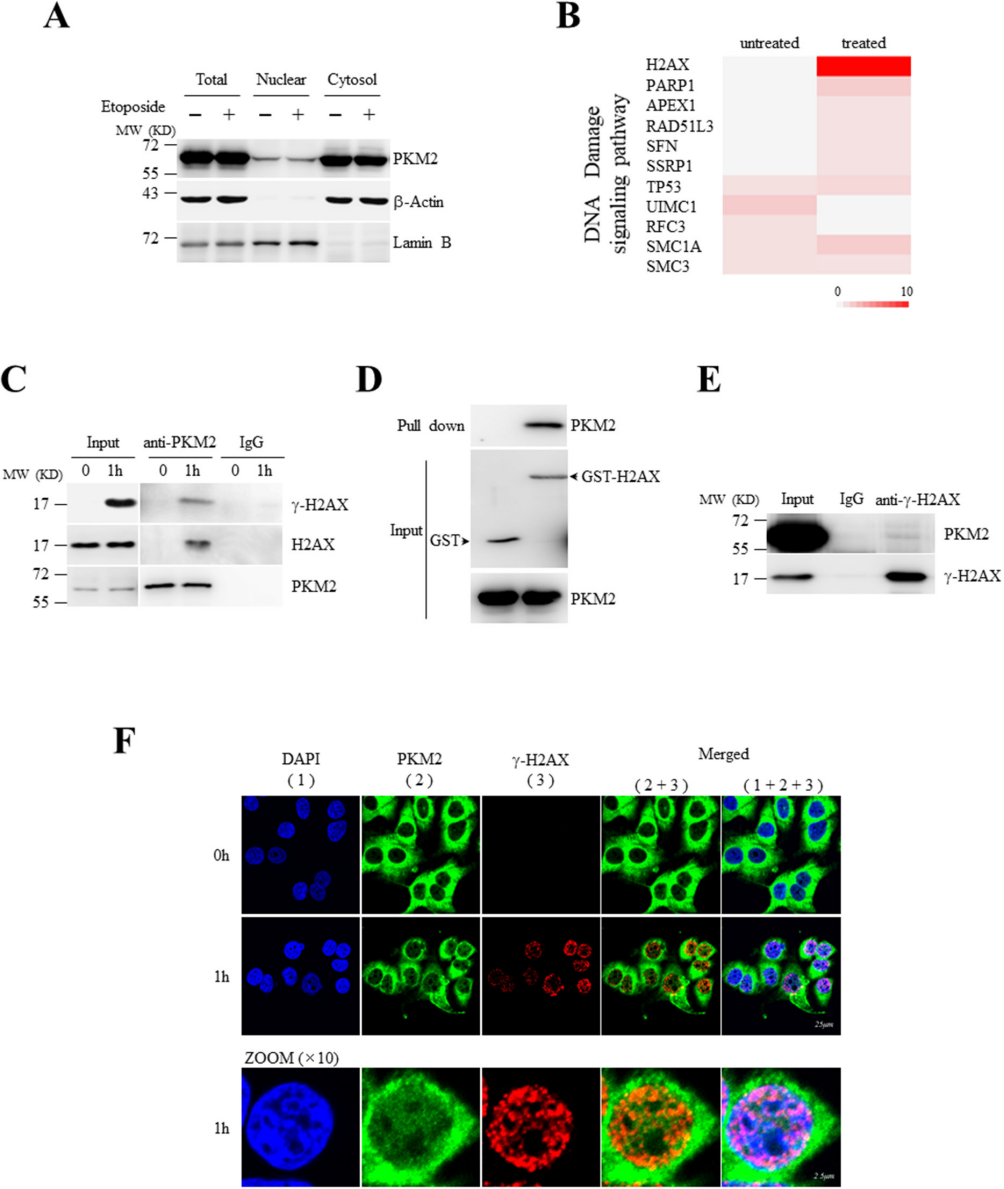
Nuclear PKM2 interacts with H2AX upon DNA damage **(A)** Cytosolic and nuclear extracts were prepared from MCF7 cells treated with or without etoposide, followed by western blot analysis for the indicated proteins. LaminB was a nuclear marker, and β-actin acted as a cytosol marker. **(B)** Heat map of gene ontology (GO) for DNA damage signaling pathway based upon the identified PKM2-interacting proteins from LC-MS/MS. GO analysis (DAVID 6.7) was applied to the specific PKM2-associated complex under etoposide treatment and untreatment for molecular function and biological process enrichment [27]. The colors in the map represent the quantitative value (normalized total spectra) according to the Scaffold_4.3.3. Color code ranges from 0 to 10. **(C)** Co-IP analysis of the interaction between endogenous PKM2 and H2AX or γ-H2AX using antibodies against PKM2 in MCF7 cells treated with or without etoposide for 1 hour. IP using rabbit IgG was a negative control. Input, 10% whole cell lysate. **(D)** GST pull-down analysis of H2AX with PKM2 using purified PKM2 and GST-tagged H2AX fusion protein. **(E)** Co-IP analysis of the interaction between endogenous PKM2 and γ-H2AX using antibodies against γ-H2AXin MCF7 cells treated with etoposide. **(F)** Immunofluorescent staining of endogenous PKM2 and γ-H2AX in MCF7 cells treated with or without etoposide for 1 hour. The bottom panel showed the amplified images for cells lined with white line. All these experiments were repeated at least for three times with the same results.

To explore the potential functions of nuclear PKM2 during DNA damage response (DDR), we previously performed immunoprecipitation-coupled liquid chromatography-mass spectrometry/mass spectrometry (LC-MS/MS) using anti-PKM2 antibody in human breast cancer cell line MCF7 and identified a number of nuclear proteins in DNA damage signaling as PKM2 binding partners, among which histone H2AX appeared (Figure 1A and 1B) [27]. Herein, we further confirmed the potential interaction of PKM2 with H2AX by co-immunoprecipitation (co-IP) based immunoblots. The results showed that anti-PKM2 antibody could pull down H2AX in MCF7 cells treated with etoposide but not untreated MCF7 cells (Figure 1B and 1C). GST-pull down assay further demonstrated a direct binding of recombinant PKM2 with GST-H2AX (Figure 1D). In response to DNA damage, H2AX is rapidly phosphorylated at serine (Ser) 139 to form γ-H2AX by phosphatidylinositol 3-kinase-related kinases (PIKK)-family kinases, serving as an early and sensitive marker for DNA double strand breaks (DSB) [35–37]. We next investigated the association of PKM2 with γ-H2AX. As depicted in Figure 1C, anti-PKM2 antibody could pull down γ-H2AX in the etoposide-treated MCF7 cells, and anti-γ-H2AX antibody also precipitated PKM2 under DNA damage conditions (Figure 1E). Moreover, immunoflurescent staining showed that nuclear PKM2 was co-localized with γ-H2AX-positive foci in MCF7 cells exposed to etoposide (Figure 1F). Collectively, these data demonstrated that PKM2 binds with H2AX in response to DNA damage stimulus.

### PKM2 promotes H2AX phosphorylation during DDR

**Figure 2:**
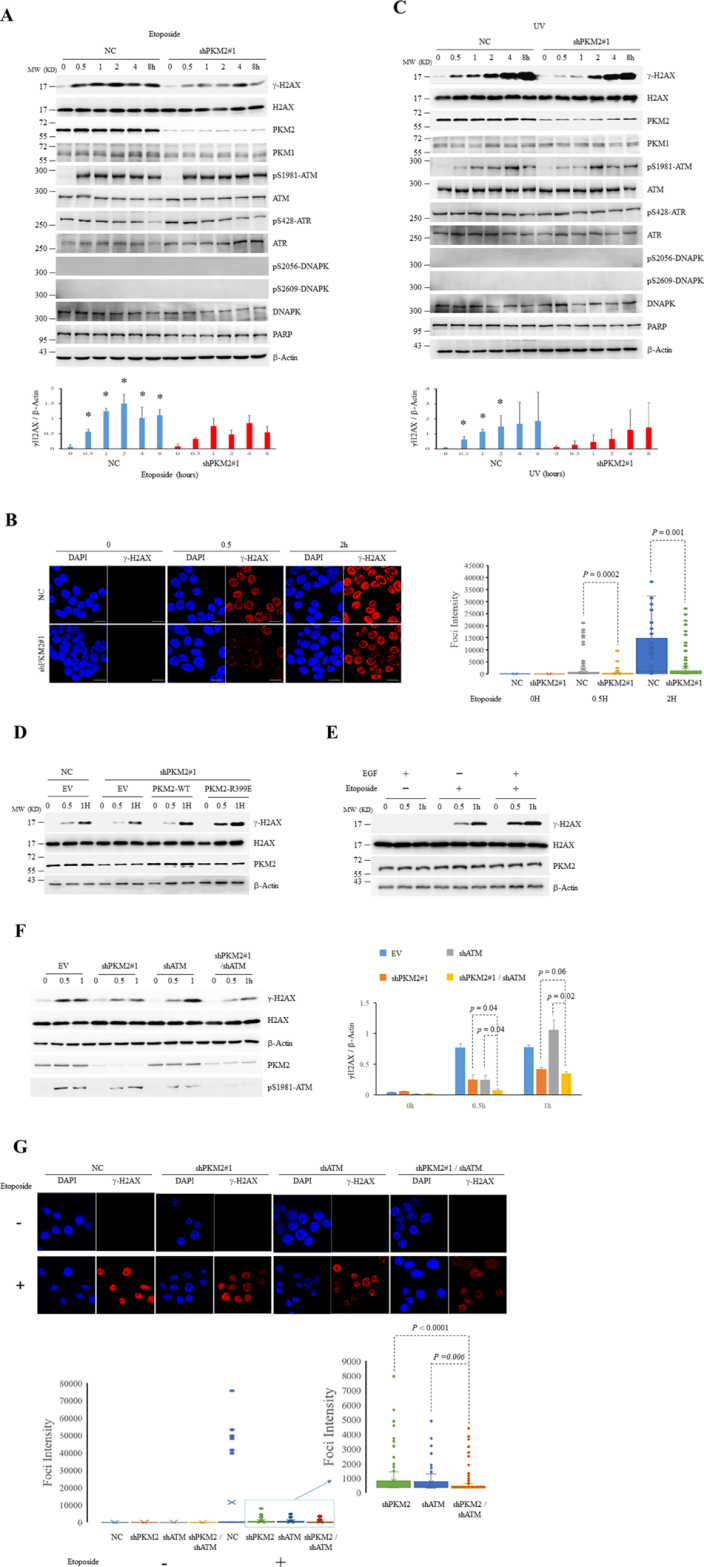
PKM2 promotes DDR **(A-C)** MCF7 cells infected with shPKM2#1 or NC were exposed to etoposide (A and B) or UV (C) for hours as indicated, followed by immunoblots for the indicated proteins (A and C) or immunofluorescent analyses (B) with DAPI co-staining. The protein bands on the gels were quantified by densitometry and shown on the bottom panels (A and C). ^*^p< 0.05 versus shPKM2. The intensity of H2AX foci was quantified by ImageJ Software and was shown on the right panel (B). **(D)** The shPKM2#1 or NC-infected MCF7 cells were transfected with empty vector (EV), wild type (WT) or R399E mutant PKM2, and treated with etoposide for the indicated hours, followed by immunoblots for the indicated proteins. **(E)** MCF7 cells exposed to EGF (100ng/ml) for 6 hours were treated with etoposide for the indicated hours, followed by immunoblots for the indicated proteins. **(F)** MCF7 cells infected with NC, shPKM2#1or/and shATM were exposed to etoposide for indicated hours, followed by immunoblots for the indicated proteins. The protein bands on the gels were quantified by densitometry and shown on the right panel. **(G)** Immunofluorescent staining of γ-H2AX in MCF7 cells infected with NC, shPKM2#1 or/and shATM treated with or without etoposide for 0.5 hour. The intensity of H2AX foci was quantified by ImageJ Software and was shown on the bottom panel. All these experiments were repeated at least for three times with the same results.

We next asked whether PKM2 would affect H2AX phosphorylation due to their interaction during DDR. To this end, a pair of small hairpin RNAs (shRNAs) specifically against PKM2 (shPKM2#1) was transfected into MCF7 (Figure 2A, Supplementary Figure 1A and 1B) and human lung cancer cell A549 (Supplementary Figure 1C), together with a non-specific shRNA as a negative control (NC). Immunoblots and immunofluorescent staining revealed that the shPKM2#1 but not NC effectively silenced PKM2 (Figure 2A and Supplementary Figure 1A-1C). Of note, the specific shPKM2#1 had no influence on PKM1 expression in MCF7 cells (Figure 2A). Then, these MCF7 cells were challenged with etoposide or irradiated by UV light. Etoposide treatment (Figure 2A and 2B) or UV irradiation (Figure 2C) rapidly and significantly induced the formation of γ-H2AX, indicating occurrence of DNA damage. Of significance, the silence of PKM2 expression remarkably inhibited both etoposide and UV treatment-induced γ-H2AX formation (Figure 2A-2C). Similar results could also be obtained in MCF7 cells transfected with another pair of shRNAs specifically against PKM2 (shPKM2#2) (Supplementary Figure 1A) and other shPKM2#1-transfected cancer cell lines such as A549 (Supplementary Figure 1C) and human leukemic NB4 cells (Supplementary Figure 1D). In line with these observations, foci of mediator of DNA damage checkpoint protein 1(MDC1), a major adaptor for transducing DNA damage and repair signal following H2AX phosphorylation [38, 39], was also diminished in response to etoposide in PKM2-depleted MCF7 cells (Supplementary Figure 1E). To confirm that the inhibitory DDR is specifically due to the silencing of PKM2, we re-introduced wild type PKM2 (PKM2-WT) into shPKM2#1-transfected MCF7 and NB4 cells. The results demonstrated that re-expressions of PKM2 could rescue etoposide-induced γ-H2AX in shPKM2#1-transfected MCF7 (Figure 2D) and NB4 cells (Supplementary Figure 1F and 1G). In addition, the rescuing effect was also conferred by PKM2-R399E mutant, a dimeric form of PKM2 (Figures 2D, Supplementary Figure 1F and 1G) [24, 25]. Furthermore, stimulating the nuclear accumulation of PKM2 by EGF treatment [2, 30] significantly enhanced etoposide-induced γ-H2AX formation (Figure 2E and Supplementary Figure 2). Collectively, these results support that nuclear PKM2 promotes H2AX phosphorylation and cellular response to DNA damage.

As mentioned above, phosphorylation of H2AX at Ser 139 in response to DNA damage is conducted by PIKK-family kinases that include ATM (ataxia telangiectasia mutated), ATR (ATM and Rad3-related) and DNAPK (DNA-dependent protein kinase). In order to elucidate whether PKM2 promotes H2AX phosphorylation through PIKK family member, we firstly detected the activation of the PIKK members using phosphorylation site-specific antibodies. As shown in Figure 2A and 2C, treatment of MCF7 cells with etoposide or UV resulted in significant activation of ATM, as determined by immunoblots using antibody selective for Ser-1981 phosphorylated ATM. In contrast, phosphorylation of ATR or DNAPK, was not apparently induced upon the exposure of etoposide or UV, in agreement with ATM being the principal kinase for γ-H2AX formation following DNA damage induced by agents that cause genotoxic stress [40]. Notably, consistent with previous reports showing that UV is different from etoposide (DNA topoisomerase II inhibitor) in triggering PIKK signals [41, 42], distinct pattern of ATM activation and γ-H2AX formation in response to etoposide and UV in MCF7 cells was observed. High level of phosphorylated ATM and H2AX was rapidly induced and maintained from 0.5 hour upon etoposide treatment in contrast to a gradually induced activation in response to UV (Figure 2A and 2C). Importantly, although depletion of PKM2 attenuated the γ-H2AX formation, it did not affect the level of Ser-1981 phosphorylated ATM (Figure 2A and 2C). Moreover, knockdown PKM2 in ATM-depleted MCF7 cells further reduced the level of γ-H2AX at 0.5 hour after etoposide treatment (Figure 2F and 2G), suggesting that PKM2 could contribute to the phosphorylation of H2AX during DDR through mechanisms other than activating ATM signals.

### PKM2 directly phosphorylates H2AX at Ser139

**Figure 3:**
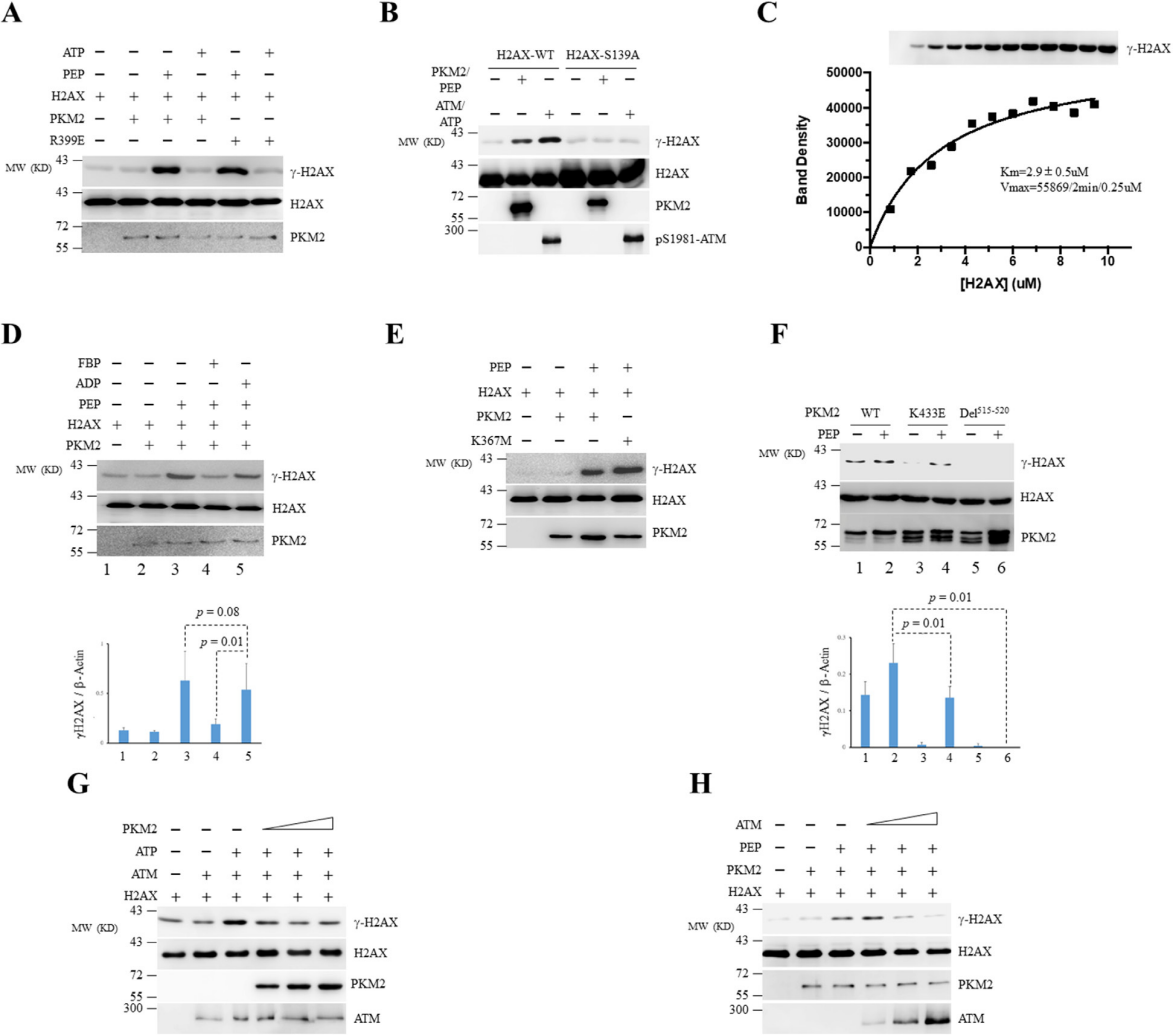
PKM2 phosphorylates H2AX at Ser139 *in vitro* **(A)** Purified recombinant PKM2-WT or PKM2-R399E mutant was incubated with recombinant GST-H2AX in the presence of PEP or ATP. H2AX, γ-H2AX and PKM2 were detected by immunoblotting. **(B)** The *in vitro* kinase reactions were performed by incubating recombinant PKM2 or ATM with WT GST-H2AX or GST-H2AX-S139A mutant in the presence of PEP (for PKM2) or ATP (for ATM). **(C)** PKM2 (0.25μM) was incubated with varied purified H2AX in the presence of excess PEP at room temperature for 2min. The reaction mixtures were then analyzed by Western blot using indicated antibodies (upper panel). The phosphorylated H2AX protein bands were quantified by densitometry and the kinetic constants were determined by fitting the data with Michaelis-Menten equation using GraphPad Prism. **(D)** Phosphorylation of GST-H2AX by recombinant PKM2 in the presence of FBP, ADP or PEP was detected by immunoblotting. **(E, F)** The *in vitro* kinase assay was carried out by mixing WT PKM2 or its mutants as indicated with GST-H2AX. The protein bands on the gels were quantified by densitometry and shown on the bottom panel (D, F). **(G, H)** Phosphorylation of recombinant H2AX by ATM or PKM2 in the presence of escalating concentration of PKM2 (G) or ATM (H) was analyzed by *in vitro* kinase assay. Immunoblots were performed for indicated proteins. All these experiments were repeated at least for three times with the same results.

Nuclear PKM2 has been reported to display protein kinase activity towards a number of substrates for which PKM2 uses PEP but not ATP as a phosphate donor [2, 23, 24, 30]. Considering that the activity of kinases known to be responsible for generation of γ-H2AX was not changed upon PKM2 depletion, we postulated that H2AX might be directly phosphorylated by PKM2. We set up *in vitro* kinase reaction and observed that recombinant PKM2 could phosphorylate H2AX in the presence of PEP [12, 24], but not ATP (Figure 3A), indicating that PKM2 presents kinase activity on H2AX with the PEP as phosphate donor. Consistent with the previous reports [43–45], recombinant ATM phosphorylated H2AX but not H2AX-S139A mutant in the presence of ATP (Figure 3B). Likewise, PKM2 failed to phosphorylate H2AX-S139A in the presence of PEP (Figure 3B), suggesting that the PKM2 and ATM phosphorylate the same serine of H2AX. Furthermore, the dimeric PKM2-R399E mutant, which was reported to still present protein kinase activity as dimers [24], also phosphorylated H2AX to a similar degree to PKM2-WT in the presence of PEP (Figure 3A).

Next, we assessed the kinetic parameters for the reaction between PKM2 and H2AX by densitometry. We used an optimized reaction condition where 0.25uM PKM2 was incubated with various concentrations of H2AX in the presence of excess PEP at room temperature. The reaction mixtures were then analyzed by Western blot. The phosphorylated H2AX protein bands were quantified by densitometry and the kinetic constants were determined by fitting the data with Michaelis-Menten equation using Graph Pad Prism. As depicted in Figure 3C, H2AX, as a substrate, could be phosphorylated by PKM2 with a Km of 2.9±0.5uM.

PKM2 is a tightly regulated enzyme which responds not only to the availability of PEP and ADP substrates, but also to FBP, a glycolysis intermediate acting as an allosteric activator of PKM2 [9, 46]. To address whether ADP or/and FBP-binding site is involved in protein kinase activity of PKM2 towards H2AX, we carried out competition kinase analysis in the presence of ADP or FBP. The results demonstrated that FBP markedly reduced PKM2-induced γ-H2AX formation (Figure 3D). In contrast, the addition of ADP did not significantly affect the phosphorylation of H2AX by PKM2, suggesting that the protein kinase active site might be distinct from the pyruvate kinase active site [47]. In line with this finding, PKM2 K367M mutant, which lost the ADP-binding ability [2], still exhibited similar protein kinase activity with PKM2-WT (Figure 3E). However, deletion of the FBP-binding pocket of PKM2 (Del^515-520^) abolished its kinase activity on H2AX (Figure 3F). Mutation of lysine 433 (K433E), which is located at the lip of the pocket [15, 48], also showed a slightly decreased kinase activity compared with its WT counterpart (Figure 3F). Because both Del^515-520^ and K433E could bind with H2AX as evaluated by GST-pull down assays (Supplementary Figure 3), we postulated that the decreased kinase activity may be due to lower kinectics of the PKM2 mutants for this reaction which required further investigation. Taken together, these results indicate that FBP-binding sites of PKM2 is critical for the protein kinase activity of PKM2.

We next detected the potential association of PKM2 with ATM on H2AX phosphorylation using *in vitro* kinase assay. As shown in Figure 3G and 3H, recombinant PKM2 and ATM interfered with the other’s kinase activity in a dose dependent manner *in vitro*. These results demonstrated that ATM and PKM2 work competitively in phosphorylating H2AX *in vitro*.

### PKM2 phosphorylates H2AX under DNA damage stimuli *in vivo*

**Figure 4:**
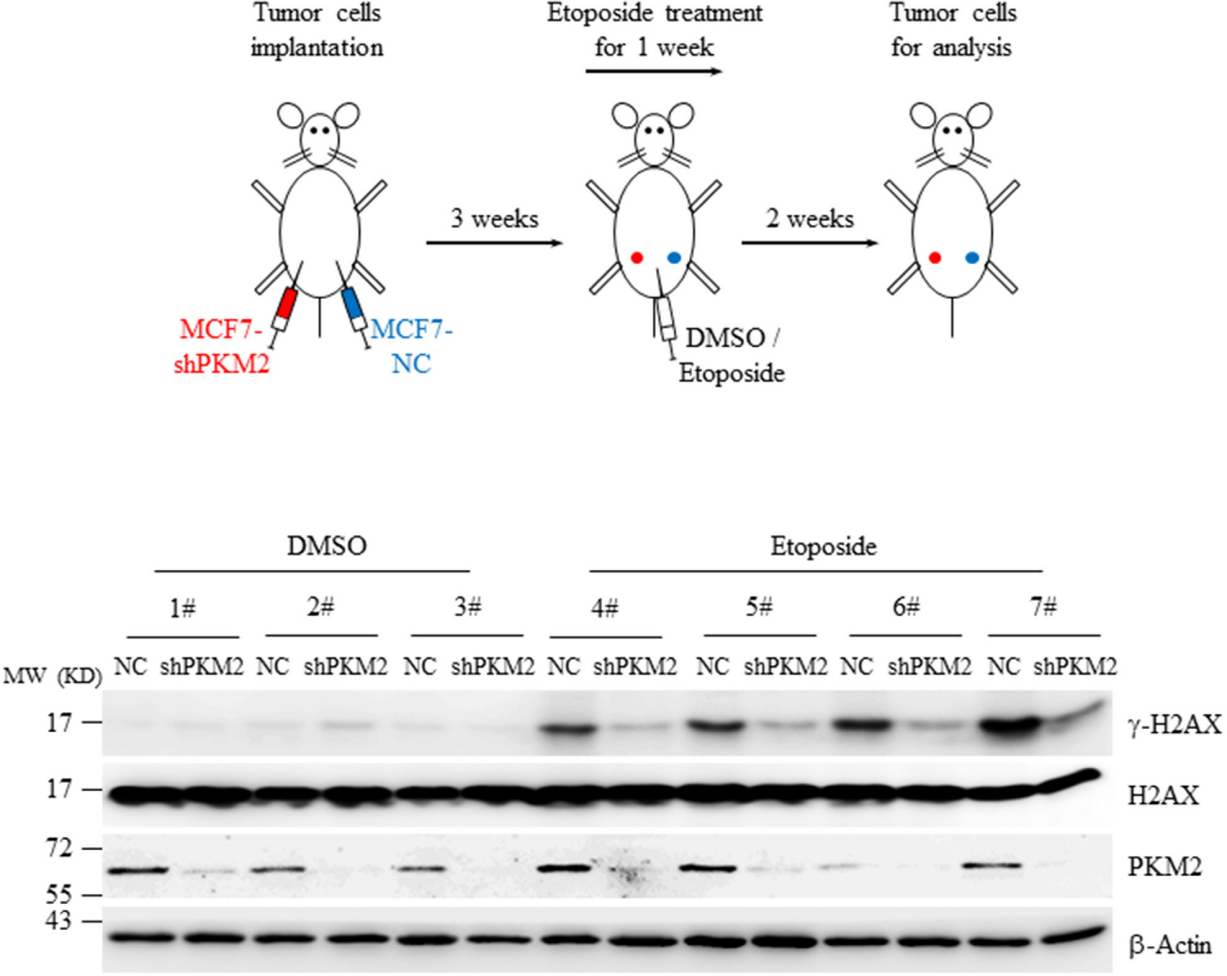
Knockdown of PKM2 decreased γ-H2AX level in xenografts MCF7 cells infected with NC or shPKM2#1 were injected into the left or right side of the mammary fat pad of nude mice and grown for three weeks. Afterwards, a dose of 0.1mg/kg of etoposide was administrated intraperitoneally for 7 consecutive days. Two weeks later, the fresh tumors from the mice were collected, homogenated and subjected to western blot analysis with the indicated antibodies.

To test whether PKM2 is a potent H2AX kinase *in vivo*, xenograft experiments followed by immunoblots analysis of γ-H2AX in the tumors were performed. MCF7 cells infected with NC or shPKM2#1 were injected subcutaneously into the mammary fat pad of the nude mice followed by etoposide treatment for 7 days. The tumors were collected two weeks afterwards and subjected to western blots. As shown in Figure 4, etoposide treatment induced significant γ-H2AX formation in contrast to etoposide-untreated group. Compare to NC tumors, administration of etoposide led to decreased γ-H2AX in shPKM2 tumors (Figure 4), demonstrating that PKM2 phosphorylates H2AX *in vivo*.

### PKM2 promotes cell growth with increased DNA damage and chromosomal aberrations following DDR

**Figure 5:**
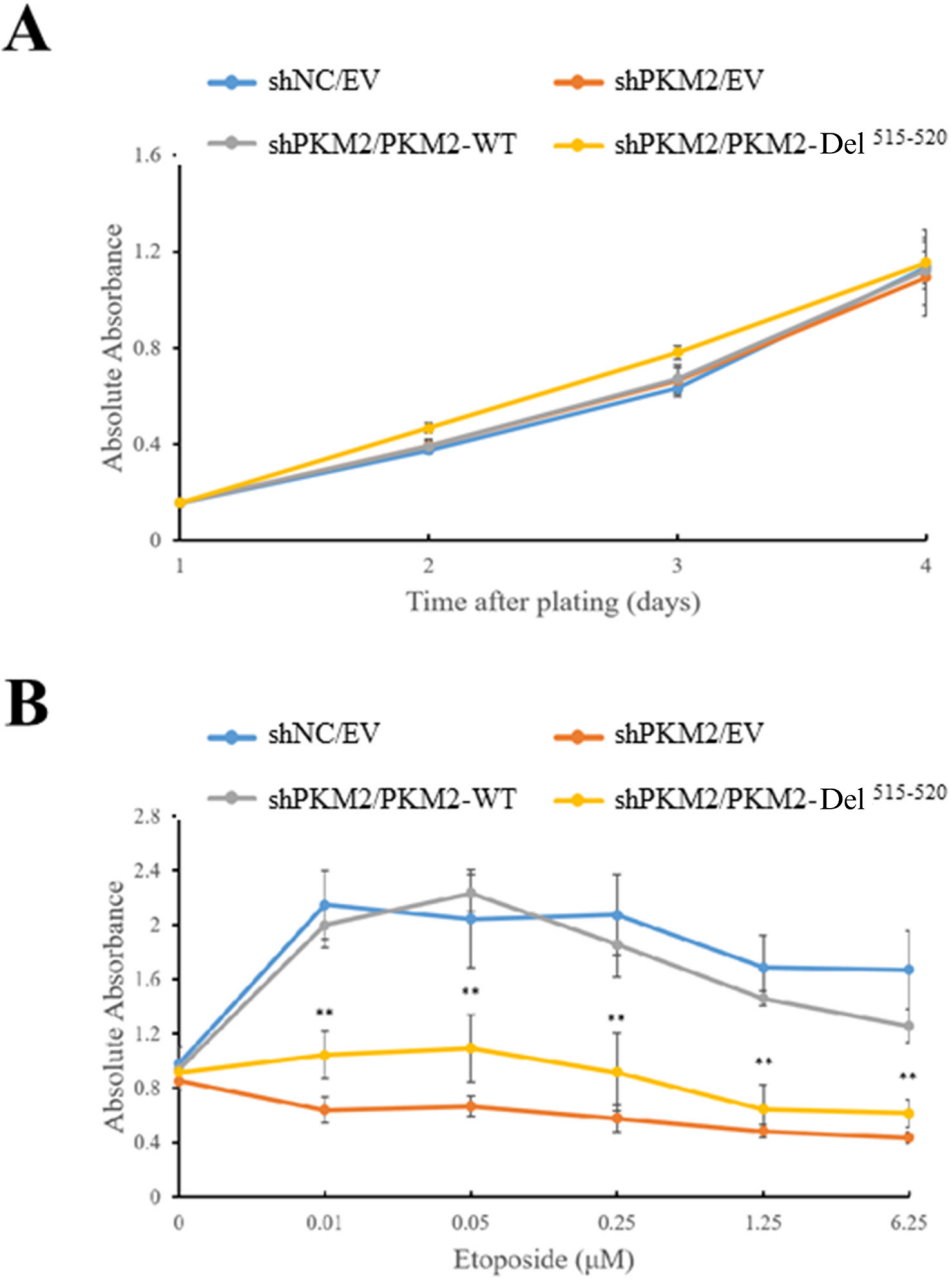
PKM2 promotes cell growth under DNA damage condition Expression of empty vector (EV), wild type (WT) PKM2 or PKM2-Del^515-520^ mutant in PKM2-depleted MCF7 cells followed by treatment with **(B)** or without **(A)** etoposide. Then the cell proliferations were measured by CCK-8 assay. ^**^p< 0.01 versus PKM2-WT.

We previously showed that PKM2 promotes tumor growth under DNA damage [27]. To further clarify the role of PKM2 protein kinase activity towards H2AX in promoting cell proliferation, we created a cell line by stable expression of Del^515-520^ in PKM2-depleted MCF7 cells. Cell counting kit-8 (CCK-8) assay showed that the PKM2 expression, whether wild type or Del^515-520^ did not cause apparent change in cell proliferation without etoposide administration (Figure 5A). However, consistent with our previous report [27], treatment of etoposide induced a decreased proliferation of PKM2-depleted MCF7, which could be rescued by expression of WT PKM2 (Figure 5B). In contrast, expression of Del^515-520^ did not fully restore the proliferation rate as WT PKM2 did (Figure 5B), suggesting that the protein kinase activity, at least partially, mediated the effects of PKM2 in promoting cell proliferation following DDR.

**Figure 6:**
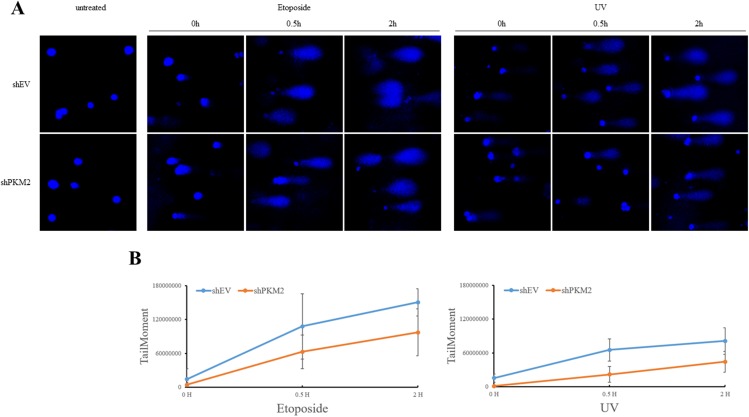
PKM2 increased chemical- and UV-damaged DNA **(A)** MCF7 cells infected with shPKM2#1 or NC were exposed to etoposide or UV for indicated hours, followed by detection of DNA damage through alkaline comet assay. **(B)** Images were analysed with an ImageJ software plugin Comet Assay from Microscopy Services Laboratory (https://www.med.unc.edu/microscopy) and levels of DNA damage were shown.

To address whether PKM2 induces higher level of damage to DNA in response to genotoxic stress, we evaluated DNA strand breaks using the comet assay, which can effectively detect single and double strand breaks in DNA under alkaline conditions [49, 50]. The results showed that etoposide and UV induced significant DNA strand breaks in a time dependent manner (Figure 6). Compared to NC cells, etoposide and UV induced decreased level of DNA strand breaks in PKM2-depleted MCF7 cells (Figure 6), indicating that PKM2 increased the damage to DNA.

**Figure 7:**
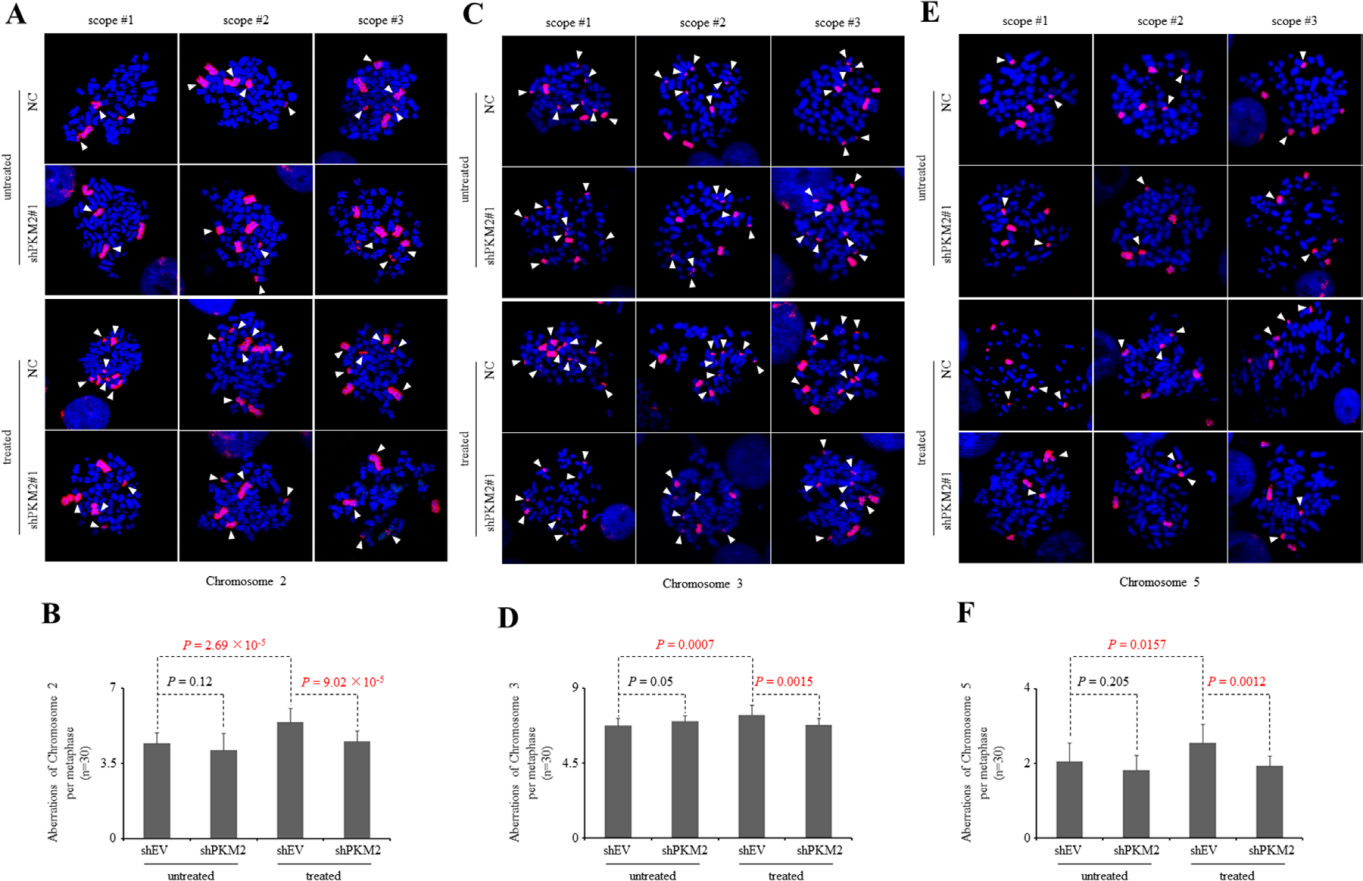
Knockdown of PKM2 reduces chromosomal aberrations following DDR **(A, C, E)** Three representative metaphase spreads of MCF7 cells infected with NC or shPKM2#1 either untreated or 24hr following etoposide treatment. Fluorescence *in situ* hybridization (FISH)-mediated whole chromosomal staining was used to identify aberrations of chromosome 2 (A), 3 (C) and 5 (E). **(B, D, F)** Quantification of total chromosome aberrations/metaphase (30 metaphases scored/group). Data are mean ± SD of triplicates in an independent experiment, which were repeated at least for three times with the same results.

**Figure 8:**
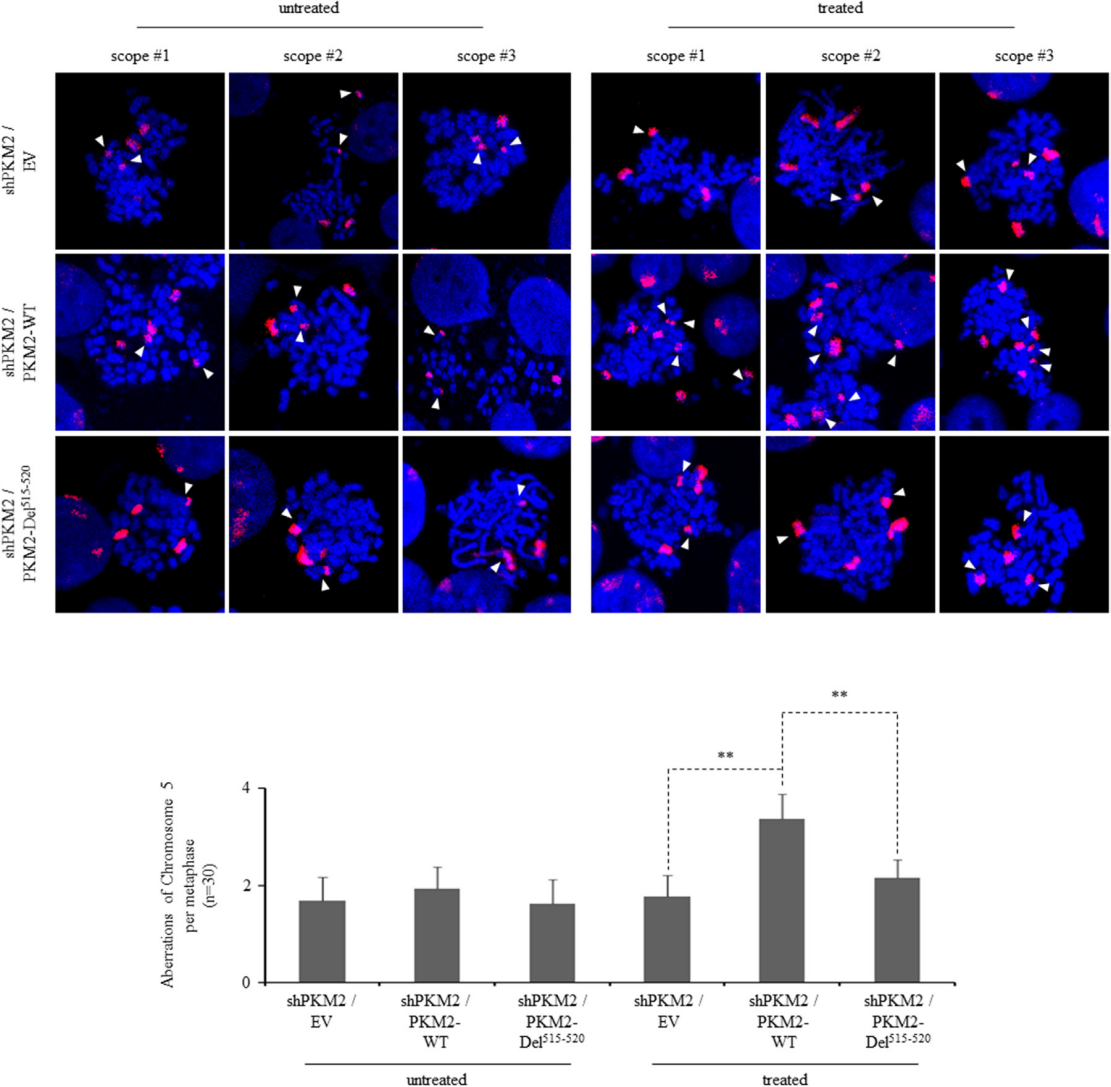
PKM2 expression increases chromosomal aberrations following DDR Three representative metaphase spreads of MCF7 cells reconstitutively expressing empty vector (EV), PKM2-WT or PKM2-Del^515-520^ mutant either untreated or 24hr following etoposide treatment. FISH-mediated whole chromosomal staining was used to identify aberrations of chromosome 5. Bottom panel shows the quantification of total chromosome aberrations/metaphase (30 metaphases scored/group). Data are mean ± SD of triplicates in an independent experiment, which were repeated at least for three times with the same results. ^**^p< 0.01 versus PKM2-WT.

Because increased rates of DNA damage if overwhelming the ability of cellular repair systems may cause genomic instability [51–53], which is a hallmark of cancer cells, we further looked into the genomic integrity in the presence or absence of PKM2 by staining chromosomes 2, 3 and 5. Our results demonstrated that knockdown of PKM2 alone did not yield significant difference in chromosomal aberrations (Figure 7). However, etoposide-treated PKM2-depleted MCF7 cells displayed a decrease in chromosomal aberrations in these three chromosomes (Figure 7) compared with NC cells, while re-expression of PKM2-WT in MCF7 cells could effectively restore etoposide-induced significant aberrations in the chromosome 5 detected (Figure 8). In contrast, PKM2-Del^515-520^mutant which lost the ability to phosphorylate H2AX (Figure 3F) failed to restore etoposide-induced chromosomal aberrations (Figure 8), indicating that the phosphorylation of H2AX by PKM2 contributes to increased genomic instability following DNA damage. Collectively, our data suggested that PKM2 promoted tumor growth with increased DNA damage which may leads to high risk of genomic instability in response to DNA-damaging agents.

## DISCUSSION

Phosphorylation of H2AX at Ser 139, generating γ-H2AX, is an early and sensitive marker of DNA DSB. It is a key step in DDR that initiates the recruitment of downstream signaling and repair proteins such as MDC1 at the break sites, where γ-H2AX foci is formed. H2AX can be phosphorylated by PIKK family proteins that include ATM, ATR and DNAPK. These three kinases distinguish in the kinetics of activation and the types of stimuli to which they respond best but function redundantly under most conditions [54]. Herein, we identified that PKM2 as a novel protein kinase targeting H2AX when cells are challenged with DNA-damaging agents. Instead of using ATP as phosphate donor like ATM, PKM2 utilizes PEP when phosphorylating H2AX. This might be of particular importance for rapid-growing tumor cells because of the inadequate blood and oxygen supply which limits the production of ATP. Furthermore, our results showed that PKM2 inhibits the kinase activity of ATM towards H2AX *in vitro*. Therefore, we propose that phosphorylation of H2AX by PKM2 to initiate DDR may act as an adaptive response in tumor cells exposed to genotoxic stress.

How the protein kinase activity of PKM2 is triggered upon DNA damage is of great interests. The functions of PKM2, whether enzymatic activity or non-metabolic role identified heretofore, is largely associated with its conformation state. Unlike PKM1 which constitutively exists as a stable tetramer, PKM2 is convertible between enzyme-active tetramer and inactive dimer. As a protein kinase, it is active in the form of dimer when phosphorylating stat3 at tyrosine 705 [24], while both dimers and tetramers can phosphorylate Bub3 at tyrosine 207 [25]. The predominant form of nuclear PKM2 is dimer rather than tetramer in MCF7 cells [27], suggesting that the dimeric PKM2 contributes to the phosphorylation of H2AX in cells. On the other hand, since the protein level of nuclear PKM2 was not changed upon DNA damage, a plausible mechanism responsible for the activation of protein kinase activity would be post-translational modifications. A number of post-translational modifications including S37-phosphorylation, Y105-phosphorylation and K433-acetylation that may modulate nuclear PKM2 function [20, 28, 55] were detected using site-specific antibodies. Neither the level of phosphorylation of PKM2 at S37, Y105 nor the acetylation at K433 was apparently changed upon etoposide treatment. However, immunofluorescent assay did show a colocalization of S37 phosphorylated PKM2 with γ-H2AX, indicating that S37 phosphorylation may participate in stimulating the protein kinase activity of PKM2 (data not shown). Further investigations should follow to identify the potential mechanism of the activation of protein kinase activity.

Genomic instability is an important hallmark of cancer which may result from increased frequency of genetic damages or interrupted DNA repair [50, 56]. We observed that expression of PKM2 endows the tumor cells with increased amount of DNA damage and chromosomal aberrations after the cells were challenged with etoposide. More importantly, replacement of PKM2 with the kinase dead mutant diminished etoposide-induced chromosomal aberrations, suggesting a role of PKM2 in modulating genomic integrity through phosphorylating H2AX. Currently there is a lack of understanding as to exactly how PKM2 controls DNA damage and genomic instability. Meanwhile, the other DDR-related PKM2-interacting proteins identified by LC-MS/MS such as APEX1 (apurinic/apyrimidinic endodeoxy ribonuclease 1) and RAD51L3 (DNA repair protein RAD51 homolog 4) may potentially provide plausible explanation and deserved to be further investigated.

Taken together, our data demonstrated that PKM2 phosphorylates H2AX in response to DNA damage. We also showed that PKM2 modulate DNA damage and genomic instability following DDR. These observations reveal a novel role of PKM2 in tumorigenesis and establish a link between glycolytic enzyme and genomic instability.

## MATERIALS AND METHODS

### Materials

Etoposide (VP16) was purchased from Calbiochem. Fructose 1, 6-bisphosphate (FBP), phosphoenolpyruvic acid (PEP), adenosine diphosphate (ADP), adenosine triphosphate (ATP), hygromycin B were purchased from Sigma. Rabbit polyclonal antibodies against PKM2, H2AX, γ-H2AX, ATM, Phospho-ATM (Ser1981), ATR, Phospho-ATR (Ser428), DNAPK and phospho-DNAPK (Ser 2056), and β-actin were obtained from Cell Signaling Technology. Phospho-DNAPK (Ser 2609) antibody was purchased from Signal way Antibody (College Park). Goat antibody against LaminB was obtained from Santa Cruz Biotechnology (Santa Cruz). Mouse antibody for γ-H2AX were from Abcam. Monoclonal antibodies for His or GST were from Sigma.

### Cell culture

Human MCF7 and A549 cells were maintained in Dulbecco's Modified Eagle's Medium (DMEM) supplemented with 10% fetal bovine serum (FBS). MCF7 cells were cultured with 0.01 mg/ml human recombinant insulin (Sigma). NB4 cells were cultured in RPMI-1640 medium (Sigma) supplemented with 10% or 20% FBS. For triggering DDR, MCF7 cells were treated with 100μM etoposide or exposed to 40 J/m^2^ UV. A549 or NB4 cells were exposed to 100 μM or 1 μM etoposide respectively.

### Vector construction

Human PKM2 cDNA was amplified from MCF7 cells by RT-PCR and then cloned into psumo3 vector provided by Dr Aiwu Zhou to construct His-tagged PKM2 protein-expressing plasmid or into pBabe vector (Clontech) to generate psumo3-PKM2 and pBabe-PKM2. All point mutants of PKM2 (R399E, K367M, K433E, Del^515-520^) were created utilizing QuickChange Site-Directed Mutagenesis Kit (Stratagene). The psumo3-H2AX were subcloned from pGEX-H2AX vector provided by Dr Huang Lei using the following primer pairs: 5’-TATGGATCCTCGGGCCGCGGCAAGAC-3’ (forward) and 5’-GTAGTCGACTTA GTACTCCTGGGAGGCC-3’(reverse). pSIREN PKM2 shRNA was generated using CATCTACCACTTGCAATTA oligonucleotide targeting transcript of exon 10 of the PKM [2]. The pSIREN of ATM shRNA target sequence was AACATACTACTCAAAGACATT (PMID: 22123827). Non-sense mutations of C1170T, C1173T, T1174C and G1176T of PKM2 [2] were cloned into the rescuing plasmids pQCXIN Retrovial (Clontech). The sequences of all cDNA inserts of plasmids were confirmed by sequencing.

### Subcellular fractionation

Cells were suspended in hypotonic buffer (10mM Tris-HCl, pH 7.9, 1.5mM MgCl_2_, 10mM KCl, 1mM DTT, 1 tablet/10ml EDTA-free complete protease inhibitor cocktail (Roche) and 1 tablet/10ml phosphatase inhibitor cocktail (Roche)) by a Dounce homogenizer (40 strokes). Nuclear pellets were separated from cytoplasm by centrifugation for 10 minutes at 1000g. The supernatants (cytoplasmic extract) were removed and transferred into new tubes. Nuclear pellets were washed with hypotonic buffer twice, and resuspended in the nuclear extraction buffer (50mM Tris-HCl, pH 7.9, 1mM MgCl_2_, 1mM DTT, 0.1% NP40, 250units/ml Benzonase (Sigma), 1 tablet/10ml EDTA-free complete protease inhibitor cocktail and 1 tablet/10ml phosphatase inhibitor cocktail) by sonication. The supernatants (nuclear extracts) were centrifuged for 10 minutes at 12,000g.

### Immunoprecipitation analysis

Whole-cell extracts were prepared in 300μl of lysis buffer (50mM HEPES, 50mM NaCl, 0.1% Tween20, 10% glycerol, 20mM sodium pyrophosphate, 1mM dithiothreitol, plus protease inhibitors) and incubated overnight with indicated antibodies (including γ-H2AX and anti-PKM2) and protein A/G plus-agarose (Santa Cruz Biotechnology) at 4°C. After IP, the beads were intensively washed with washing buffer (50 mM Tris-HCl, pH 7.6; 300 mM NaCl; 1 mM EDTA; 0.5% NP-40; 10% glycerol). Then the precipitates were analyzed by Western blot.

### Western blots

Protein extracts were equally loaded on 10-12% SDS-polyacrylamide gel, and transferred to nitrocellulose membrane (Amersham Bioscience). The blots were stained with 0.2% Ponceau S red to ensure equal protein loading. After blocking with 5% nonfat milk in PBS, the membrane was incubated with indicated antibodies, followed by horseradish perioxidase (HRP)-linked secondary antibodies (Cell Signaling Technology). Detection was performed by chemiluminescence phototope-HRP kit (Cell Signaling Technology). The protein bands on the gels were quantified by densitometry. Scanning was performed at optimal exposure time where band intensity was proportional to the concentration of protein present. Gel photographic images were stored as GRAYSCALE pictures in the TIF format and were processed using ImageJ Software.

### Confocal microscopy

Cells were treated as described in the text, harvested on slides and fixed. After permeabilization with 0.1% (v/v) Triton X-100 in PBS and blocking with 2% (w/v) BSA in PBS, cells were incubated overnight with the indicated antibodies followed by secondary antibodies conjugated either with Alexa Fluor 555 dye (Invitrogen) or Alexa Fluor 488 dye for 1 h. Cellular DNA was counterstained with 4, 6-diamidino-2-phenylindole (DAPI; Molecular Probes). Fluorescence signals were detected on a Nikon A1R confocal laser microscope.

### Purification of recombinant proteins

His-tagged WT and mutant PKM2 as well as GST-tagged H2AX protein were expressed in bacteria BL21 (DE3) by induction with 0.5mM isopropylthiogalactopyranoside (IPTG) at 30°C and purified from the cytosol of the expressed cells by affinity chromatography on Ni-NTA-agarose (Qiagen).

### Size-exclusion chromatography

The bacterially purified PKM2 protein or the nuclear lysates (from 5-7 mg/ml of total protein), was loaded into a Superdex 200 10/300GL column and eluted with elution buffer (50 mM phosphate, 0.15M NaCl, pH 7.2). Each 300μl consecutive fraction was collected, and 20μl of each fraction was analyzed by immunoblot.

### *In vitro* kinase assay

Bacterially purified WT and mutants of His-tagged recombinant PKM2 (10μg/ml) were incubated with GST-tagged H2AX (10μg/ml) in 50ul kinase buffer (Cell Signaling Technology) at 25°C for 2 hours. The reactions were terminated by addition of SDS loading buffer and heated to 100°C. The reaction mixtures were then analyzed by SDS-PAGE.

### Kinetic determination of PKM2 and H2AX interaction

To determine the kinetic parameters ( K_m_) values for PKM2. The kinase reactions were performed using fixed PKM2 concentration (0.25μM) with varied substrate (H2AX) in the presence of excess PEP at room temperature for 2min. The reaction mixtures were then analyzed by Western blot. The phosphorylated H2AX protein bands were quantified by densitometry and the kinetic constants were determined by fitting the data with Michaelis-Menten equation using Graph Pad Prism.

### Cell transfection

For transient transfection, Lipofectamine 2000 transfection reagent was used following the manufacturer's protocol (Invitrogen). Retroviruses were prepared by transient co-transfection with helper plasmids into 293T cells using Lipofectamine 2000. After the transfection, 1 μg/ml of puromycin was added to screen stable cell lines for further assays.

### Comet assay

A single-cell gel electrophoresis (comet assay) kit was employed for evaluating DNA damage (Trevigen, Inc.). Etoposide- or UV-treated or untreated MCF7 cells were resuspended in PBS and combined with molten low melting agarose at a ratio of 1:10 (v/v) and immediately pipetted 50 μl onto CometSlide. After cell lysis, samples were treated with NaOH solution (300 mM NaOH, 1mM EDTA) and applied to electrophoresis. Then the samples were stained with DAPI, and viewed by a fluorescence microscopy [50]. Images were analysed with an ImageJ software plugin Comet Assay from Microscopy Services Laboratory (https://www.med.unc.edu/microscopy) [57].

### Metaphase spreads and chromosomal aberration analysis

MCF7 cells (WT or PKM2-depleted) were treated with or without etoposide for 4 hours followed by releasing for 24 hours. The cells were then incubated with Colcemid (0.2ug/ml) for 4 hours after which cells were trypsinized and spun down. The cell pellets were resuspended with hypotonic solution (75mM KCl) for 10 min at room temperature, and then fixed by freshly prepared Carnoy solution (3:1 v/v methanol/aceic acid) [58]. Metaphases were hybridized with whole chromosomes 2, 3, 5 fluorescence-labelled DNA probe (XCP, Whole-Chromosome Probe, MetaSystems) as described in the manual, and were counterstained with DAPI. The slides were viewed with Nikon A1R confocal laser microscope (100×). Chromosome aberrations were determined by its structural incomplete, such as translocation, exchange, rearrangement, fragment, or break.

### Xenograft tumor assay

The 6- to 8-week-old female nude mice (Slaccas Laboratory Animal, Shanghai, China) were supplemented with 0.36 mg of 60-day release 17β-estradiol pellets (Innovative Research) by subcutaneous inoculation. Then, 1 × 10^6^ MCF7 cells infected with NC or shPKM2#1 was injected into the left (NC) or right (shPKM2) side of the mammary fat pad of the mice. After 3 weeks, a dose of 0.1mg/kg of etoposide or DMSO was administrated intraperitoneally for 7 consecutive days. Two weeks afterwards, the tumors were collected and subjected to western blot analysis.

### Statistical analysis

The Student’s t-test was used to compare the difference between two different groups. A value of p<0.05 was considered to be statistically significant.

## SUPPLEMENTARY MATERIALS FIGURES


