# Supplementary material for: Pyruvate kinase M2 phosphorylates H2AX and promotes genomic instability in human tumor cells
Source: Oncotarget. 2017 Nov 17;8(65):109120–34. doi: 10.18632/oncotarget.22621 (PMC5752508; doi:10.18632/oncotarget.22621)
Supplement: Supplementary file 1 [file oncotarget-08-109120-s001.pdf]

Pyruvate kinase M2 phosphorylates H2AX and promotes genomic instability in human tumor cells

SUPPLEMENTARY MATERIALS

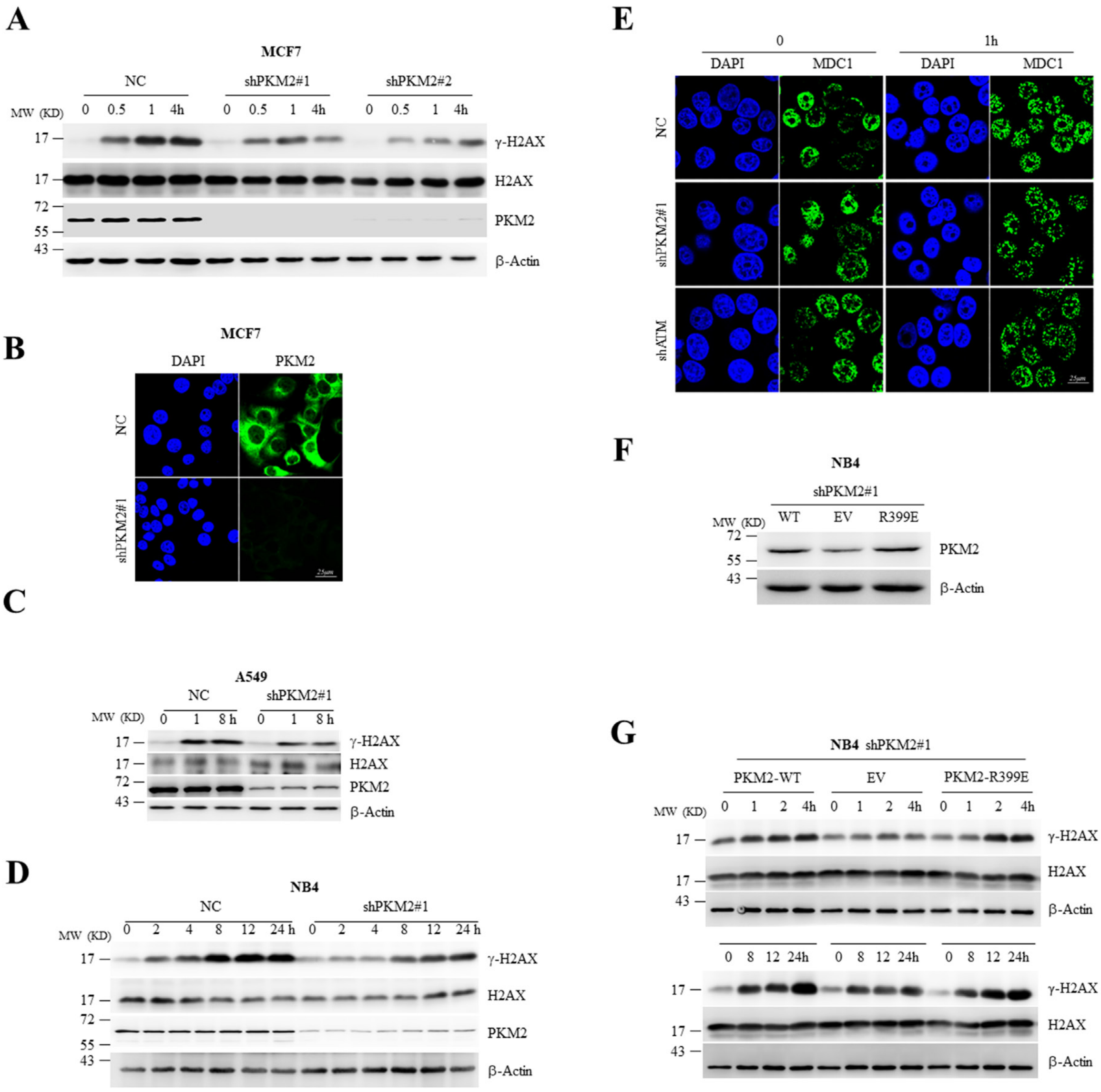

Supplementary Figure 1: PKM2 promotes DDR in cancer cell lines. **(A)** MCF7 cells infected with NC, shPKM2#1 or shPKM2#2 were exposed to etoposide for the indicated hours (h). Immunoblotting was performed for the indicated proteins. **(B)** Immunofluorescent staining of PKM2 in NC or shPKM2#1-infected MCF7. **(C)** Western blotting analysis of extracts prepared from the NC and shPKM2#1-infected A549 cells exposed to etoposide for the indicated hours. **(D)** Western blotting analysis of extracts prepared from the NC and shPKM2#1-infected NB4 cells exposed to etoposide for the indicated hours. **(E)** Immunofluorescent staining of MDC1 with nuclear re-staining in NC, shATM or shPKM2#1-infected MCF7 cells with or without treatment of etoposide for 0.5 hour. **(F, G)** The shPKM2#1-infected NB4 cells were transfected with wild type (WT), empty vector (EV) or R399E mutant PKM2 (F), and treated with etoposide for the indicated hours, followed by immunoblots with the indicated antibodies (G).

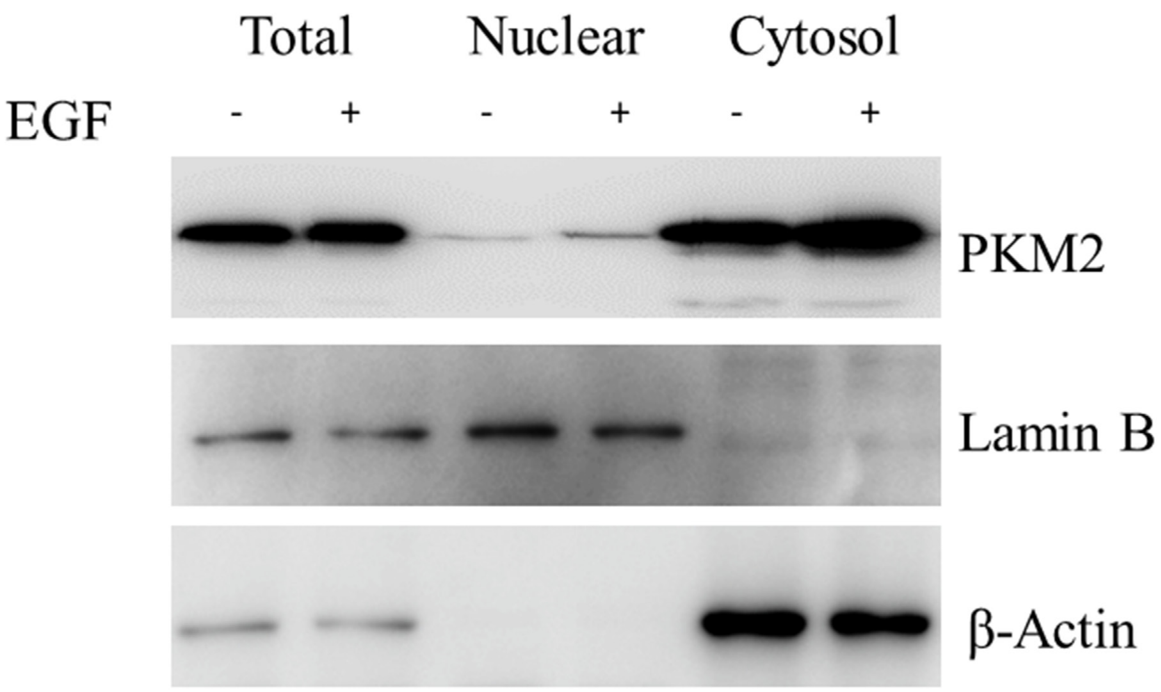

Supplementary Figure 2: MCF7 cells were exposed to EGF (100ng/ml) for 6 hours followed by subcellular fractionation and immunoblots for the indicated proteins.

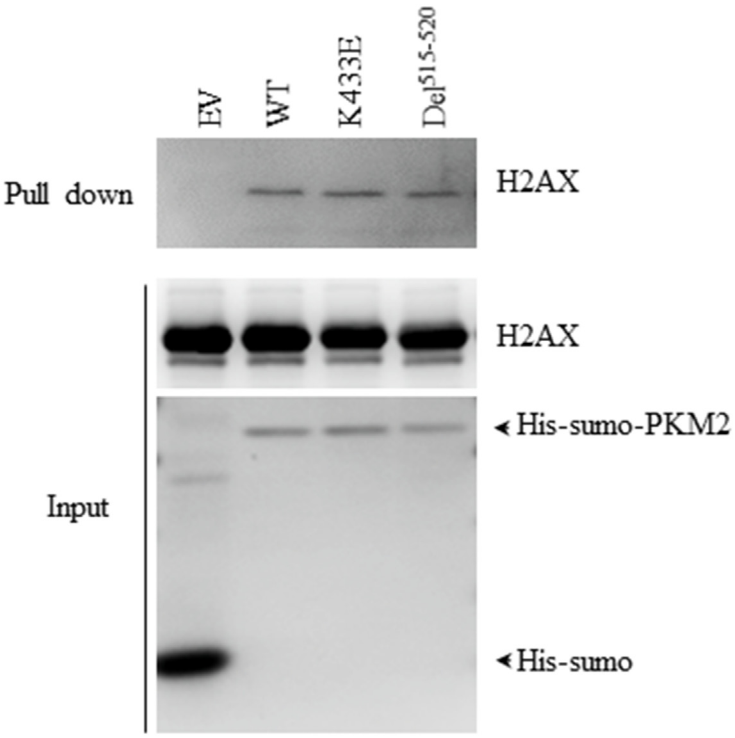

Supplementary Figure 3: GST pull-down analysis of H2AX with WT PKM2 or PKM2 mutants using purified His-tagged PKM2 and GST-tagged H2AX fusion protein.
